# Metabolic Engineering of Yeast for the Production of 3-Hydroxypropionic Acid

**DOI:** 10.3389/fmicb.2018.02185

**Published:** 2018-09-20

**Authors:** Rong-Yu Ji, Ying Ding, Tian-Qiong Shi, Lu Lin, He Huang, Zhen Gao, Xiao-Jun Ji

**Affiliations:** ^1^College of Biotechnology and Pharmaceutical Engineering, Nanjing Tech University, Nanjing, China; ^2^School of Pharmaceutical Sciences, Nanjing Tech University, Nanjing, China; ^3^State Key Laboratory of Materials-Oriented Chemical Engineering, Nanjing Tech University, Nanjing, China; ^4^Jiangsu National Synergetic Innovation Center for Advanced Materials, Nanjing Tech University, Nanjing, China

**Keywords:** 3-Hydroxypropionic acid, metabolic engineering, yeast, malonyl-CoA reductase pathway, β-alanine pathway

## Abstract

The beta-hydroxy acid 3-hydroxypropionic acid (3-HP) is an attractive platform compound that can be used as a precursor for many commercially interesting compounds. In order to reduce the dependence on petroleum and follow sustainable development, 3-HP has been produced biologically from glucose or glycerol. It is reported that 3-HP synthesis pathways can be constructed in microbes such as *Escherichia coli, Klebsiella pneumoniae* and the yeast *Saccharomyces cerevisiae*. Among these host strains, yeast is prominent because of its strong acid tolerance which can simplify the fermentation process. Currently, the malonyl-CoA reductase pathway and the β-alanine pathway have been successfully constructed in yeast. This review presents the current developments in 3-HP production using yeast as an industrial host. By combining genome-scale engineering tools, malonyl-CoA biosensors and optimization of downstream fermentation, the production of 3-HP in yeast has the potential to reach or even exceed the yield of chemical production in the future.

## Introduction

With the reduction of easily obtainable fossil fuels and sustainable development becoming the mainstream of the world, petroleum-based compounds are gradually being replaced by bio-based compounds to meet the increasing demands for bulk materials and fine chemicals alike (Nielsen and Keasling, 2016; Chen et al., 2018a). Increasingly sophisticated cell factories are being built to produce many renewable bio-based chemicals. Among them, 3-hydroxypropionic acid (3-HP) ranked third in the top 12 value-added chemicals from biomass listed by the United States Department of Energy in 2004 (Werpy et al., 2004). As a hydroxy-functionalized carboxylic acid, 3-HP is a promising platform chemical, which can be transformed to a variety of worthy products such as malonic acid, acrylic acid, acrylic esters, 1,3-propanediol, and building blocks for biodegradable polymers (Liu et al., 2017). The two most important present applications of 3-HP are the production of acrylic acid and poly(3-hydroxypropionic acid) (P[3-HP]). Acrylic acid is able to be obtained by dehydrating 3-HP, and acrylic acid-derived products are widely used in paints, paper, baby diapers, adhesives, textiles, specialty coatings, and superabsor. While P[3-HP] has good mechanical properties and can be hydrolyzed enzymatically, mainly used for making surgical products (Zhang et al., 2004; Pina et al., 2011). It has been pointed out that 3-HP has a wide market and great application value. It can be obtained by chemical synthesis and biosynthesis methods. Traditional chemical synthesis of 3-HP is well-established, but resource limitations, high price and the toxicity of the used raw materials do not meet the new sustainable development goals. Compared with chemical synthesis, biosynthesis methods have many advantages, including cheaper substrates, mild reaction conditions and simple operation, which are compatible with sustainable development, and the reduced amounts of byproducts help reduce the cost of production.

In recent years, the biosynthesis of 3-HP has attracted wide attention, which led to rapid new developments. First discovered as an intermediate metabolite of the 3-HP cycle in *Chloroflexus aurantiacus*, 3-HP was also found in *Acidianus brierleyi*, *Acidianus ambivalens*, and *Sulfolobus metallicus* (Holo, 1989; Strauss et al., 1992; Jiang et al., 2009).

Up till now, three known pathways for 3-HP production have been constructed in recombinant organisms, the glycerol pathway, the β-alanine pathway, and the malonyl-CoA reductase (MCR) pathway. The natural generation of 3-HP proceeds through the glycerol pathway (Lan et al., 2015). However, the intermediate metabolite 3-hydroxypropionaldehyde (3-HPA) is toxic to cells and requires an efficient aldehyde dehydrogenase to convert it to 3-HP. To prevent the accumulation of this toxic intermediate, current emphasis is on balancing the enzymes’ expression (Jiang et al., 2009). The MCR pathway, which used the intermediate malonyl-CoA as the substrate, only involves one enzymatic reaction, mainly depending on MCR (Chen et al., 2014; Lian et al., 2018). However, the β-alanine pathway is considered to be the most economically attractive (Borodina et al., 2015).

Three common hosts for the biosynthesis of 3-HP from glucose and/or glycerol are *Escherichia coli*, *Klebsiella pneumoniae*, and the yeast *Saccharomyces cerevisiae*. Although *E. coli* is the most widely studied, there are many limitations slowing further research. In *E. coli*, the conversion of glycerol to 3-HP requires to add additional coenzyme B_12_ to the culture medium, which is sensitive to oxygen and replaced by vitamin B_12_ because of the aerobic conditions required for 3-HP production (Roth et al., 1996; Daniel et al., 1998; Toraya, 2002; Rathnasingh et al., 2012; Kumar et al., 2013; Chen et al., 2014; Ko et al., 2014). Since *E. coli* cannot produce coenzyme B_12_, it requires exogenous addition, which increases the cost of industrial production significantly (Valdehuesa et al., 2013). *K. pneumoniae* is another alternative host, and it can synthesize coenzyme B_12_ naturally (Kumar et al., 2013). However, in addition to being a pathogenic bacterium, it is also a challenge for *K. pneumoniae* to balance electron transportation from NADH to NAD^+^ with the oxygen demand and the decrease of coenzyme B_12_ production under aerobic conditions (Kumar et al., 2013; Chen and Nielsen, 2016). Moreover, the pH decrease due to the production of 3-HP in *E. coli* and *K. pneumoniae* leads to an acidic intracellular pH, which seriously affects cell growth, resulting in reduced 3-HP tolerance. The toxicity of 3-HP requires the addition of a large amount of base titrant into the cultures when using these bacteria. By contrast, yeast has a strong intrinsic tolerance to low pH, and recombinant strains based on yeast are not affected by 3-HP toxicity, significantly reducing the fermentation cost (Liu et al., 2017). Kildegaard et al. (2014) proposed that 3-HP could be converted to toxic 3-HPA by aldehyde dehydrogenases and subsequently detoxified through glutathione-dependent reactions in yeast. Due to its advantages of high tolerance to relatively low pH, available whole-genome sequences of multiple strains, and easy molecular genetic manipulation, yeast is perhaps the most promising host strain for 3-HP production (Liu et al., 2017).

## Engineering Yeast for Efficient 3-HP Production

**FIGURE 1:**
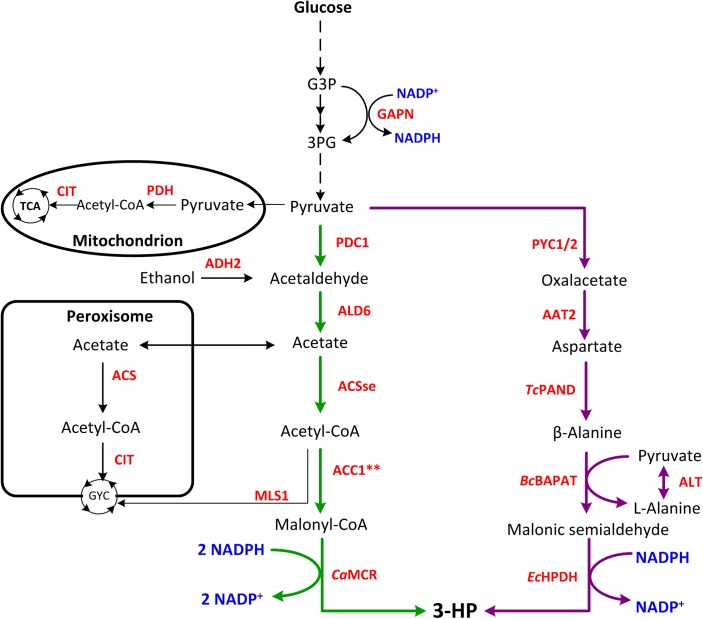
Metabolic pathways constructed in yeast for 3-HP production. Malonyl-CoA reductase pathway is shown in green, and β-alanine pathway is shown in purple. G3P, glyceraldehyde 3-phosphate; 3PG, 3-phosphoglycerate; GYC, glyoxylate cycle; TCA, tricarboxylic acid cycle; AAT2, aspartate aminotransferase 2; ACC1^∗∗^, Snf1 deregulated acetyl-CoA carboxylase (two site mutations, one is Ser659→Ala, the other is Ser1157→Ala); ACS, acetyl-CoA synthetase; ACSse, acetylation-insensitive acetyl-CoA synthetase from *Salmonella enterica*; ADH2, alcohol dehydrogenase 2; ALD6, NADP-dependent aldehyde dehydrogenase; ALT, alanine aminotransferase; *Bc*BAPAT, β-alanine-pyruvate aminotransferase from *Bacillus cereus*; CIT, citrate synthase; *Ec*HPDH, 3-hydroxypropionate dehydrogenase from *E. coli*; GAPN, NADP-dependent G3P dehydrogenase; *Ca*MCR, malonyl-CoA reductase from *Chloroflexus aurantiacus*; MLS1, cytosolic malate synthase; PDC1, pyruvate decarboxylase; PDH, pyruvate dehydrogenase complex; PYC1/2, pyruvate carboxylase 1/2; *Tc*PAND, aspartate decarboxylase from *Tribolium castaneum*.

As shown in **Figure 1**, two efficient pathways for 3-HP production from glucose have been constructed in yeast – the MCR pathway and the β-alanine pathway, and the main metabolic engineering strategies are summarized in **Table 1**.

**Table 1:** Metabolic engineering strategies for 3-hydroxypropionate production in yeast.

Metabolic engineering strategies	Beneficial effect for 3-HP production	Reference
Increasing the supply of malonyl-CoA: Overexpression the enzymes (ALD6, ACSse, ADH2) for acetyl-CoA accumulation; Deleting the MLS1 to block the consumption of acetyl-CoA; Overexpression the ACC1 catalyzing acetyl-CoA to malonyl-CoA. Increasing the supply of NADPH: Overexpression the GAPN catalyzing the formation of extra NADPH. Establishing the MCR pathway: Overexpression the *Ca*MCR.	3-HP production was increased to 463 mg/L	Chen et al., 2014
Generating the ACC1 mutant (ACC1Ser659Ala Ser1157Ala) through mutating the potential phosphorylation sites to abolish the post-translational regulation Increasing the ACC1 activity by overexpression of the ACC1 mutant	3-HP production was up to ∼2.2-fold more than that of the wild-type ACC1	Shi et al., 2014
Constructing the β-alanine pathway by overexpressing AAT2, PYC1, PYC2, ALT, BcBAPAT, EcHPDH, and multiple copies of TcPAND	13.7 g/L 3-HP was generated through the constructed β-alanine pathway from glucose in fed-batch fermentation at low pH.	Borodina et al., 2015
Increasing the supply of acetyl-CoA by overexpressing *PDC1*, *ALD6*, and ACSse; Engineering the cofactor specificity of the GAPN to increase the production of NADPH at the expense of NADH and thus improve 3-HP production and reduce formation of glycerol as by-product; Integrating multiple copies of *Ca*MCR and ACC1 mutant genes into the genome.	3-HP was produced at a titer 7.37 g/L in a carbon-limited fed-batch fermentation	Kildegaard et al., 2016
Developing a malonyl-CoA biosensor based on the bacterial transcription factor FapR to monitor and precisely control the intracellular malonyl-CoA concentration	3-HP titer was enhanced i by 120%	Li et al., 2015
A hierarchical dynamic control strategy to control the expression level of *Ca*MCR depending on the intracellular malonyl-CoA concentration: The upper level of control was to dynamically downregulate fatty acid biosynthesis using HXT1 promoter. The lower level was based on the malonyl-CoA biosensor.	3-HP production was increased by 10-fold	David et al., 2016
Improving the availability of malonyl-CoA through down-regulating lipid synthesis. Manipulating the phospholipid synthesis transcriptional regulators including Ino2p, Ino4p, Opi1p, and a series of synthetic Ino2p variants, combining with studying the inositol and choline effect.	3-HP production was increased by 9-fold	Chen et al., 2017
Identifying and characterizing promoters that depend on glucose concentration for use as dynamic control elements. Identifying 34 candidate promoters that strongly responded to glucose presence or absence. A subset of promoters, pADH2, pICL1, and pHXT7, were demonstrated as suitable for dynamic control of 3-HP production.	Regulating the 3-HP pathway by the ICL1 promoter resulted in 70% improvement of 3-HP titer in comparison to PGK1 promoter.	Maury et al., 2018

*AAT2, aspartate aminotransferase 2; ACC1, acetyl-CoA carboxylase; ACSse, acetylation-insensitive acetyl-CoA synthetase from *Salmonella enterica*; ADH2, alcohol dehydrogenase; ALD6, NADP-dependent aldehyde dehydrogenase; ALT, alanine aminotransferase; *Bc*BAPAT, β-alanine-pyruvate aminotransferase from *Bacillus cereus*; *Ec*HPDH, 3-hydroxypropionate dehydrogenase from *E. coli*; GAPN, NADP-dependent G3P dehydrogenase; *Ca*MCR, malonyl-CoA reductase from *Chloroflexus aurantiacus*; MLS1, cytosolic malate synthase; PDC1, pyruvate decarboxylase; PYC1/2, pyruvate carboxylase 1/2; *Tc*PAND, aspartate decarboxylase from Tribolium castaneum.*

### The Malonyl-CoA Reductase Pathway

Recently, the MCR pathway has been successfully constructed in yeast. This pathway relies on the conversion of glucose into pyruvate by glycolysis. The pyruvate is used to generate acetaldehyde by PDC (pyruvate dehydrogenase complex), then acetate is formed by oxidation of ADH (aldehyde dehydrogenase) and further converted to acetyl-CoA by ACS (acetyl-CoA synthetase) (Tang et al., 2015). As the most important precursor of 3-HP production, malonyl-CoA is generated from acetyl-CoA by ACC1 (acetyl-CoA carboxylase), and the final product 3-HP is produced via a two-step reaction catalyzed by MCR. MCR is a bi-functional enzyme, consisting of two short-chain domains, the N-terminal domain encoding an alcohol dehydrogenase and the C-terminal encoding an aldehyde dehydrogenase. The production of 3-HP was successfully increased by balancing these two enzyme domains (Hügler et al., 2002; Liu et al., 2013, 2016). The production of malonyl-CoA is strictly regulated in yeast, and its yield in the cytoplasm directly affects the 3-HP production. Therefore, it is meaningful to increase the malonyl-CoA concentration in the cytoplasm.

#### Improving the Supply of the Precursor Acetyl-CoA

Malonyl-CoA is an important substrate for the production of 3-HP by MCR. It is mainly produced from acetyl-CoA by ACC1, and known as a flux-controlling step (Tehlivets et al., 2007; Chen et al., 2018b). Therefore, the accumulation of acetyl-CoA in the cytoplasm has a significant impact on the synthesis of 3-HP. In *S. cerevisiae*, pyruvate in the cytoplasm can be converted to acetaldehyde through decarboxylation, thus further generate acetyl-CoA. And the acetaldehyde can further converted to ethanol by alcohol dehydrogenase, resulting in the so-called Crabtree effect (Hoek et al., 1998; Chen et al., 2013). Studies of acetyl-CoA metabolism have shown that it is quite complex in yeast, where it can be produced in the mitochondria, peroxisomes, and the cytosol (Pronk et al., 1996; Chen et al., 2012). In *S. cerevisiae*, acetyl-CoA cannot be transported to different compartments spontaneously, and instead utilizes a carnitine/acetyl-carnitine shuttle or the glyoxylate cycle (Chen et al., 2013). Therefore, many studies have focused on increasing the amount of acetyl-CoA in the cytoplasm.

Three strategies have been proposed to increase the accumulation of acetyl-CoA in the cytoplasm. The first strategy relies on pulling carbon into the cytosolic pool (Kocharin et al., 2012; Chen et al., 2013). Ethanol is the main metabolite of *S. cerevisiae*, but a by-product in the production of 3-HP. When the endogenous ADH2 (alcohol dehydrogenase) was co-expressed with ALD6 (NADP-dependent aldehyde dehydrogenase), ACC1 and a codon optimized ACS variant, ACSse (an acetylation-insensitive acetyl-CoA synthetase from *Salmonella enterica*), the flux was successfully redirected from ethanol to acetyl-CoA in the cytosol, which improved 3-HP production to 210 mg/L, up to twofold of the control strain (Chen et al., 2014). It is reported that intracellular acetyl-CoA levels could be increased to 2.19 fold by overexpression of ACS1 (acetyl-CoA synthetase 1) in *S. cerevisiae*, and improved to 5.02 fold by overexpression of ACS2 (acetyl-CoA synthetase 2) (Chen et al., 2013). ACC1 activity is repressed by Snf1 protein kinase at the protein level, as phosphorylation by Snf1 inactivates ACC1 (Woods et al., 1994). Shi et al. (2014) improved the activity of ACC1 in *S. cerevisiae* by abolishing the Snf1-dependent regulation through mutating the target sites of Snf1-mediated phosphorylation (Ser659→Ala, and Ser1157→Ala). Further overexpression of the mutated ACC1 (ACC1^∗∗^) increased the 3-HP titer to ∼279 mg/L, increased more than 2.2-fold in comparison with the wild-type.

The second strategy relies on reducing the consumption of acetyl-CoA in the cytoplasm by other accessory metabolic pathways. Acetyl-CoA is produced in four different compartments of the *S. cerevisiae* cells by different metabolic mechanisms. Pyruvate from the cytoplasm generates acetyl- CoA by PDH and then enters the tricarboxylic acid (TCA) cycle via CIT (citrate synthase). In the peroxisomes, acetyl-CoA is generated from acetate by ACS and then enters the glyoxylate cycle via CIT. Part of the cytoplasmic acetyl-CoA also enters the glyoxylate cycle via MLS (malate synthase) (Nielsen, 2014). To minimize the loss of acetyl-CoA in the cytoplasm, CIT2 and MLS1, which encode the key enzymes of the glyoxylate cycle, were individually knocked out (Chen et al., 2012, 2013). Based on the over-expression of ACC1, ACSse, ADH2 and ALD6, the 3-HP titer could reach 249 mg/L when combined with deletion of CIT2, and reach 270 mg/L when combined with deletion of MLS1 (Chen et al., 2014).

The last strategy entails constructing new acetyl-CoA pathways in the cytoplasm. The pyruvate in the cytoplasm is partially converted into acetate by PDC (pyruvate decarboxylase), partly enters the mitochondria to produce acetyl-CoA via the PDH. Although the PDH is only present in the mitochondria, if there is no MTS (mitochondrial targeting sequence) in the structural genes, PDH in the mitochondria will be redirected to the cytoplasm (cytoPDH), thus constructing a new pathway to generate acetyl-CoA directly from pyruvate. In one study, an MTS-free PDH structural genes from *S. cerevisiae* was used in combination with the PDH from *Enterococcus faecalis* to successfully increase the content of acetyl-CoA for the production of n-butanol (Lian et al., 2014).

#### Improving the Supply of the Cofactor NADPH

NADPH is another important precursor, functioned as the cofactor, for 3-HP production through the MCR pathway. In this pathway, 2 mol NADPH was needed to convert malonyl-CoA to 3-HP by MCR (Hügler et al., 2002; Valdehuesa et al., 2013; Lynch et al., 2016). It has been shown that the availability of NADPH for the production of 3-HP can be increased effectively by heterologous expression of a non-phosphorylating NADP-dependent glyceraldehyde-3-phosphate dehydrogenase (GAPN) (Bro et al., 2006; Guo et al., 2011). Moreover, although the expression of GAPN can improve the content of NADPH, it has no effect on the production and biomass yield (Verho et al., 2003; Chen et al., 2014). When the expression of GAPN was coupled with improved precursor supply, the 3-HP titer was promoted to 463 mg/L (Chen et al., 2014).

#### Precisely Control the Supply of Malonyl-CoA

Malonyl-CoA is the direct precursor of 3-HP in the MCR pathway. It is strictly regulated in yeast and its concentration is maintained at a low level. Therefore, increasing the cytosolic malonyl-CoA content is extremely important for improving the production of the malonyl-CoA derived 3-HP.

To better manipulate malonyl-CoA levels, monitoring and enhancing the content of malonyl-CoA in real time is vital. In one approach, a malonyl-CoA sensor was developed based on the FapR transcription factor from *Bacillus subtilis* by Li et al. (2015). They designed the sensor using a codon-optimized bacterial transcription factor FapR and its corresponding operator fapO to sense intracellular malonyl-CoA levels in *S. cerevisiae* for the first time (Li et al., 2015). They combined the biosensor with a genome-scale cDNA overexpression library, identifying two key genes that affect the levels of malonyl-CoA, TPI1 and PMP1. Overexpression of the key genes increased the malonyl-CoA concentration, further enhancing the 3-HP production by 120%. This sensor provides a novel way to study the complex malonyl-CoA metabolism and to enhance the 3-HP production. Utilizing the malonyl-CoA sensor, David et al. (2016) established a hierarchical dynamic pathway control system on the genetic level for 3-HP production in *S. cerevisiae*. They integrated two different levels of dynamic control. The upper level of control was used to dynamically downregulate fatty acid biosynthesis using the HXT1 promoter to modifying the FAS1 (fatty acid synthase 1) gene expression characteristics. The lower level was based on the malonyl-CoA biosensor, which was used to link the expression levels of MCR to the intracellular malonyl-CoA concentration. This novel approach significantly improved the production of 3-HP 10-fold in yeast. Chen et al. (2017) successfully improved the availability of malonyl-CoA through down-regulating the lipid synthesis. They manipulated the transcriptional regulators for phospholipid synthesis, including Ino2p, Ino4p, Opi1p, and a series of synthetic Ino2p variants, to control the malonyl-CoA levels and increase the synthesis of malonyl-CoA-derived products. The engineered strain in which multiple regulators were manipulated showed a 9-fold increase of the 3-HP titer. The study thus provides a novel strategy to regulate the intracellular malonyl-CoA availability and the 3-HP production. Maury et al. (2018) identified and characterized the yeast promoters that depend on glucose concentration, i.e., they are strongly responded to glucose presence or absence, and tried to use them as dynamic control elements for malonyl-CoA derived 3-HP production.

### The β-Alanine Pathway

Another 3-HP synthesis pathway constructed in yeast is the β-alanine pathway (**Figure 1**). Researchers from Cargill Inc. evaluated three synthetic pathways for 3-HP production which utilize β-alanine as an intermediate metabolite (Liao et al., 2007; Gokarn et al., 2011). Two of these are completely unfeasible in thermodynamic terms, while the third pathway is relatively short and appeared feasible. However, due to the relatively complex synthesis of β-alanine by the organism itself, the efficiency of 3-HP synthesis remained very low. Nevertheless, many studies have optimized this pathway and improved the production of 3-HP in yeast (Burk and Osterhout, 2010). This way, glucose is converted into pyruvate through glycolysis, which in turn is used to generate β-alanine in three steps. The β-alanine is derived from aspartate by the action of PAND (aspartate decarboxylase), which is encoded by the pabD gene in bacteria but does not exist in yeast.

Jessen et al. (2015) proposed that the transformation of β-alanine to malonic semialdehyde relies on GABT (γ-aminobutyrate transaminase) and BAPAT (β-alanine-pyruvate aminotransferase). Subsequently, malonic semialdehyde is further dehydrogenated by HPDH (3-hydroxypropionate dehydrogenase or 3-hydroxyisobutyrate dehydrogenase) to yield 3-HP (Borodina et al., 2015). In the whole pathway, the NADPH produced by glycolysis is later consumed by HPDH, and just 1 mol ATP is generated (Kumar et al., 2013). Therefore, to improve the 3-HP production through the β-alanine pathway must resort to the heterologous enzymes. The BAPAT activity has a large influence on the production of 3-HP. *Bc*BAPAT (β-alanine-pyruvate aminotransferase from *Bacillus cereus*) is considered to be the most active enzyme, comparing favorably with the BAPAT homologs from *Pseudomonas putida* and *P*. *aeruginosa* (Liao et al., 2007, 2010). *Ec*HPDH (3-hydroxypropanoate dehydrogenase from *E. coli*) is another efficient enzyme for the conversion of β-alanine into 3-HP. Aspartate-1-decarboxylases from different sources were also tested to enable β-alanine biosynthesis from aspartate, among which *Tc*PAND (aspartate-1-decarboxylase from *Tribolium castaneum*) is the most efficient (Lian et al., 2018). Overexpression of endogenous PYC (pyruvate carboxylase) and AAT2 (cytoplasmic aspartate aminotransferase) can be used to effectively channel the flux from pyruvate to aspartate. Overexpression of 5 genes, PYC1, PYC2, *Bc*BAPAT, *Tc*PAND, and *Pp*HIBADH (NADH-dependent 3-hydroxyisobutyrate dehydrogenase from *P. putida*) yielded ∼0.6 g/L and ∼0.49 g/L 3-HP from glucose and xylose, respectively (Kildegaard et al., 2015). A 3-HP high-producing strain can be obtained by overexpressing five genes, AAT2, *Bc*BAPAT, *Ec*HPDH, and PYC1/2, together with multiple copies of *Tc*PAND, producing 12.2 g/L and 13.7g/L of 3-HP by deep fermentation and controlled fed-batch fermentation at pH 5.0 with glucose as the substrate, respectively (Borodina et al., 2015).

## Conclusion and Perspectives

The biosynthesis of 3-HP has developed rapidly in recent years, and two synthetic pathways of 3-HP, the MCR and β-alanine pathways, have been established in yeast by rational metabolic engineering. Using the MCR pathway, the yield of 3-HP could be increased up to ∼10 g/L by increasing the supply of the precursors acetyl-CoA and NADPH, as well as optimizing the medium and culture conditions. Similarly, the yield of 3-HP in the β-alanine pathway reached 13.7 g/L in the controlled fed-batch fermentations at pH 5.0 by increasing the supply of the precursors β-alanine and NADPH. Although both approaches can yield over 10 g/L of 3-HP when implemented in yeast, this is still far from the high titers needed for the commercially viable production of commodity chemicals, which typically starts at 100 g/L (Kumar et al., 2013). Malonyl-CoA biosensors and genome-scale engineering tools can be combined to improve the 3-HP production in yeast. Nevertheless, although yeast is an intrinsically acid-tolerant host, which greatly simplifies the downstream process, developing an effective and economical 3-HP downstream purification technology could also improve the output of 3-HP after separation and purification (Li et al., 2015; Si et al., 2015, 2017; David et al., 2016; Bao et al., 2018). Further optimization of the recombinant yeast strains and development of more powerful separation techniques could raise the titer and yield of 3-HP enough to enable economically competitive bioproduction at an industrial scale in the future.

## Author Contributions

R-YJ, YD, ZG, and X-JJ wrote the manuscript. T-QS, LL, and HH assisted with writing, editing, and finalizing the manuscript.

## Conflict of Interest Statement

The authors declare that the research was conducted in the absence of any commercial or financial relationships that could be construed as a potential conflict of interest.
